# Evaluation of critical risk factors in the implementation of modular construction

**DOI:** 10.1371/journal.pone.0272448

**Published:** 2022-08-08

**Authors:** Hamza Pervez, Yousaf Ali, Dragan Pamucar, Mónika Garai-Fodor, Ágnes Csiszárik-Kocsir

**Affiliations:** 1 School of Management Sciences, Ghulam Ishaq Khan Institute of Engineering Sciences & Technology, Topi, KPK, Pakistan; 2 Department of Operations Research and Statistics, Faculty of Organizational Sciences, University of Belgrade, Belgrade, Serbia; 3 Keleti Károly, Faculty of Business and Management, Óbuda University, Budapest, Central Hungary, Hungary; Sichuan Agricultural University, CHINA

## Abstract

Modular construction is considered as a preferred construction method over conventional construction due to a number of benefits including reduction in project completion time, improved environmental performance, better quality, enhanced workers’ safety and flexibility. However, successful implementation of modular construction is hindered by various risk factors and uncertainties. Therefore, it is imperative to perform a comprehensive risk assessment of critical risk factors that pose a negative impact on the implementation of modular construction. Moreover, there is also a relatively less rate of modular construction adoption in developing countries, highlighting the need to focus more on underdeveloped regions. This study aims to propose a risk assessment framework for identification, evaluation and prioritization of critical risk factors affecting the implementation of modular construction in Pakistan. 20 risk factors were identified from previous literature which were then evaluated to shortlist the most significant risks using Fuzzy Delphi. The most significant risk factors were then prioritized using a novel Full-Consistency Method (FUCOM). The results specified ‘Inadequate skills and experience in modular construction’, ‘Inadequate capacity of modular manufacturers’ and ‘Inability to make changes in design during the construction stage’ as top three critical risks in the implementation of modular construction. This is the first study to propose a risk assessment framework for modular construction in Pakistan. The results of the study are useful to provide insights to construction industry practitioners in highlighting and eliminating risks involved in modular construction planning and execution.

## Introduction

Construction sector plays a crucial role in the progression of a country’s economy. One of the major roles of construction sector is to fulfil the infrastructure and housing needs of countries. Currently, cast in-situ or on-site construction is the most commonly used construction method across the globe. However, this traditional method poses various challenges, a few of them being on-site safety concerns, longer project completion time, cost over-runs, decreased quality, increased construction waste, inefficiency and environmental sustainability concerns [1–3]. Many developed countries are now shifting their focus towards innovative construction techniques to resolve the aforementioned issues in conventional construction method. In this regard, off-site construction plays a significant role in resolving the current issues in conventional on-site construction. It refers to a process in which the building elements and structures are manufactured in off-site locations and then transported to construction site for final assembly and installation [4, 5]. According to the percentage of work performed in a factory, off-site construction can be categorized into four types namely component subassembly, Non-volumetric preassembly, Volumetric preassembly, and Modular construction [6, 7]. Modular construction is the most complete form of off-site construction in which 80–85% of work involved in the construction stage is carried out in a factory whereas the remaining tasks such as excavation, laying of foundation and module installation are performed at the construction site [8]. The benefits associated with modular construction are workflow continuity [9], reduction in construction waste [10, 11], reduction in project completion time [12, 13], reduction in carbon emissions [14, 15], reduction in number of on-site contractors and improved workers’ safety [16, 17].

The relative advantages of modular construction over traditional construction have led to its increased adoption in many countries. Australian construction firms have identified modular construction as a key vision for the improvement of current state of construction industry [18, 19]. In UK, the percentage of homes built from modular prefabricated methods is around 30% whereas in Scotland and Ireland, the rate of prefabrication method of construction is expected to increase up to 70% in the coming years [20, 21]. Moreover, in Asia, Japan is the leading practitioner of off-site modular construction with construction firms producing approximately 70,000 prefabricated homes every year. Similarly, Malaysian construction firms have also given importance to the use of modular construction practices. Industrialized Building System (IBS) is a terminology commonly used in Malaysia, which is defined as a construction method in which elements of a building are manufactured in a factory environment, and then transported and assembled at on-site, thereby minimizing additional site-work [22]. In China, conventional building construction accounts for a huge amount of construction waste and carbon footprint. With the application of off-site construction methods, 74% reduction in construction waste and 34% reduction in carbon emissions can be achieved [11, 23].

Considering the increased adoption of modular construction due to its well documented benefits specifically project completion time, cost and construction waste reduction [6, 10, 12], other less developed countries can also use it as a sustainable alternative over conventional construction. However, the adoption and implementation of modular construction on a large scale is a complicated process associated with multiple risks and uncertainties. For less developed countries, modular construction is an innovative construction method with a unique scope, steps and interfaces [24, 25]. These unique characteristics are associated with various complexities which give rise to potential risks hereby posing novel challenges to decision makers. For example, the supply chain of modular construction consists of multiple upstream and downstream segments which requires effective coordination among the involved project participants including developer, client, main contractor, sub-contractors, designer, architect, manufacturer and transporter of modular building [26]. The increased number of supply chain segments as well as the complexity of material and information flow may result in disruptions which can ultimately led to decline in schedule performance and project delays [27]. Modular building is an engineer-to-order product which means that shortages cannot be compensated by other third-party manufacturers [28, 29]. Furthermore, the transportation of modular components to site require special vehicles for transportation and cranes for assembly. The restrictions on the size and weight of modular components puts constraints on the delivery of components to construction site [30]. From an economic point of view, the initial capital cost for implementing modular construction is high and uncertainty in demand creates a risk of delay in achieving return on initial investment [31]. Due to the novelty of modular construction in developing countries, lack of experience and skilled workforce is also a potential risk in the implementation of modular construction [32].

The aforementioned risks eventually pose detrimental impacts on the schedule performance, quality, safety and the productivity of modular construction projects. Therefore, it is necessary to risk assessment of potential risks in order to reduce their negative impacts on construction projects [33]. Developed countries have vast experience in handling modular construction risks as a consequence of their failures and success in the construction industry. On the contrary, developing countries have inadequate experience in this field; hence, the impact of risks on the implementation of modular construction in developing countries is more significant as compared to developed countries. The identification and prioritization of risks based on their impact on construction projects can help practitioners recognise the most critical risks and subsequent risk management strategies can be formulated for the mitigation of identified risks. Previous literature on modular construction risks in the context of developing countries is somewhat limited. A study carried out in Malaysian construction industry assessed various risks in modular construction from a contractor’s point of view [34]. The study identified financial constraints, unresolved contractual issues, lack of equipment and skilled labour, and defective designs as the topmost risks in modular construction from contractors’ perception. Similarly, another study modelled risks in the transition towards modular construction in China to determine the mutual influence of identified risks [35]. The study highlighted insufficient policies and regulations, lack of expertise and knowledge in modular construction techniques, low standardization and dominance of traditional construction as the major risk factors.

The construction industry of Pakistan faces problems in the completion of construction projects on time resulting in project delays [36, 37]. In addition to this, cost overruns, quality issues and inability to meet project success factors also have a damaging impact on project completion [38, 39]. The offsite modular construction practices have proven their worth in terms of quicker project delivery and better quality as compared to conventional construction [6]. Though, very limited applications of modular construction are witnessed in the Pakistani construction industry. A research study highlighted various benefits and challenges in the implementation of offsite construction in Pakistan from the perspective of contractors and consultants [40]. However, a comprehensive risk assessment framework in the implementation of modular construction is missing. To fulfil this knowledge gap, this study aims to identify and evaluate critical risks in the implementation of modular construction in Pakistan. The identified risks are then prioritized based on the criticality of each risk when compared with each other. The most significant risks can then be mitigated by proposing appropriate strategies and policies can be formulated to assist practitioners and decision makers in the promotion of modular construction on a large scale. Since modular construction in Pakistan is in its preliminary phase, a comprehensive risk assessment framework can guide potential modular construction practitioners about how to effectively tackle potential risks and uncertainties arising during modular construction implementation. Furthermore, this research will also contribute to the academic literature by providing a detailed literature review and risk analysis of modular construction in developing country’s perspective. In this way, future studies can incorporate important findings of this research as a benchmark for risk prioritization and developing subsequent risk mitigation strategies in offsite modular construction. The remaining study is structured as follows: The next section i.e. Literature review describes an overview of modular construction and literature review on critical risks in modular construction. In the following section i.e. Methodology, the identified risks from literature review are then validated in Pakistani context through experts’ opinion and a questionnaire survey is used to examine the risks. Afterwards, the ranking of most critical risks is performed and results are described in Results and Discussion Section. Finally, in the Conclusion section, the study is concluded by highlighting certain limitations in the study and potential future research areas.

## Literature review

### Overview of modular construction

Modular construction is an innovative construction method that has revolutionized the entire construction industry by changing the way projects are planned, designed, implemented and managed [25, 41]. It is defined as an innovative construction method in which three-dimensional modular units of a building are manufactured and assembled in a factory, and transported in sections to the project site for final installation [39]. Due to substantial modifications in the design, engineering, construction and delivery of building projects, modular construction is termed as an innovation in construction [42]. The stages in the modular building construction process include design, legal approvals, procurement of construction materials, module fabrication, transportation of modular components to project site and on-site installation [43]. Owing to the manifold stages involved, modular construction supply chain is highly fragmented where several stakeholders have their own values, goals and objectives. Normally, a client contracts designer and engineers to develop a modular building design considering various factors such as reliability, durability, safety, constructability and transportability [6]. The modular building design is then forwarded to manufacturer and modules are fabricated in a controlled factory environment according to engineering specifications [44]. After fabrication, the modules are either stored temporarily in warehouses or shipped directly to project site for installation [26]. Finally, the installation process is carried out by sub-contractors at the project site according to assembly plan and layout.

The Literature on modular construction is increasing from the last few years considering its advantages over conventional construction. Research has proven that it is an environmental-friendly and cleaner approach. A study found that the implementation of modular construction has resulted in less pollution, lowered business disruptions and decreased noise related aspects of conventional construction [16, 45]. Similarly, another study reported that the increased utilization of modular construction has resulted in a noteworthy decline in the construction waste [46]. Conventional construction methods primarily drive various types of pollution, which subsequently result in negative consequences, one of them is the climate change [47]. Modular construction as an alternative construction method will leverage environmental sustainability and consequently lead towards the prevention and mitigation of climate change. To support this claim, a case study in China identified that 32 kg/m^2^ of greenhouse gas emissions (GHG) are reduced by the implementation of modular construction [48]. Moreover, a study carried out in Hong Kong identified that 52% reduction in construction waste has been achieved by increased level of prefabrication [49]. This signifies that wider adoption of modular construction can resolve the ever-rising issue of environmental sustainability. A similar research analysed and compared the amount of waste generated from modular construction compared to the conventional caste-in-situ construction. The results revealed that 83.2% construction and demolition waste reduction was achieved in the case of modular construction [50].

Along with the environment related benefits of off-site construction, Similar studies have reported other important benefits of using off-site construction methods. A research study conducted in UK perspective identified that standardization and industrialization in construction industry must be increased in order to reduce project completion time, reduce the total cost of the project and to enhance the quality of the final product [5]. This was also supported by another study, which highlighted that modular construction enhances productivity [51]. A study focusing on performance assessment of modular buildings in Australia highlighted that a 25% decrease in labour cost and a 40%-time reduction is achieved in the construction phase of modular vs conventional building construction [52]. A review study on IBS implementation in Malaysian perspective also highlighted reduced waste, improved quality, easy installation, reduced waste on construction sites, flexibility, less labour requirement and fast completion time as the key advantages of modular construction [53]. Workplace safety is another important aspect in the construction industry due to hazardous working conditions. Apart from time reductions and quality enhancement, modular construction also promises a pleasing working environment for construction workers [54, 55]. Another study examined the workplace safety issues in conventional and modular construction methods and found that relatively less injuries and fatalities have been reported in modular construction as opposed to conventional method of construction [56]. This clearly supports that modular construction methods improve workplace safety.

### Critical risk factors in modular construction

Previous literature provides useful evidence on the presence of risk factors in various construction projects. Though some risk factors are mutual in several construction projects, other risk factors are different for every construction project due to different requirements such as resources, technologies and management skills [57]. Similarly, projects involving modular construction method have specific risks. Modular construction requires significant amount of initial investment which has been highlighted in several studies [6, 18, 55, 58]. This initial investment incorporates various costs such as cost of land, factory, equipment, machinery and labour etc. Due to fragmented and interdependent segments in the supply chain, high level of integration and coordination is required for successful project delivery. The complexity of supply chain in modular construction is subjected to risk of ineffective coordination among various project participants, leading to design defects, change orders, inefficiency and schedule delays [9, 26, 59].

Stakeholders involved in the modular construction projects are also from diverse backgrounds with each having own set of value system and objectives. Unlike traditional construction where all stakeholders mutually transfer project risk to a client, project participants in modular construction are amalgamated into a single market hence responsible for steady project delivery [60]. Due to the diversity of stakeholders, conflict of interest is a significant risk which may affect the quality and performance of modular projects. Another important risk is the limited expertise and experience in modular construction, highlighted in various research studies [24, 61–64]. Limited expertise can result in problems such as poor design, manufacturing, assembly, installation and erection practices [65]. Moreover, lack of experience can also result in various conflicts due to poor coordination between designers and manufacturers in the preliminary stages of construction, problems during production and schedule delays [66].

Modular construction being a novel construction approach in various countries lacks proper design codes and standards [35, 67, 68]. This carries a since risk since implementing a novel approach in the absence of regulatory guidance and policies may result in financial loss. Lack of government support is also a significant factor since many countries have government owned construction firms as clients. In countries such as Hong Kong, China, Australia, Malaysia, and UK, government plays a crucial role in setting policies and future plans for promotion of modular construction as well as providing concessions and remittances to small and medium sized firms [18, 63]. In the absence of government support, potential risks such as difficulty in acquiring planning approvals and low market demand can restrict implementation of modular construction [35].

Other important risk factors affecting the implementation of modular construction are transportation constraints [45, 46, 55, 58], change order due to defective design [9, 34, 69], complexity of modular building design [34, 70], unskilled labour [67, 71] and technology incompetence [14, 46, 65]. A research study modelled the key risks affecting cost and schedule performance of modular construction and showed that poor logistics, delayed design changes, inept scheduling, contractual risks and inexperienced laborers are the key risks influencing modular construction cost and schedule performance [72]. Similarly, a recent research study identified construction cost, inadequate warehousing capacity and end-user preferences as the key risk factors pertinent to the implementation of modular construction [73].

While previous studies have highlighted individual risks, few studies have made an attempt to prioritize risk factors. A study was carried out in identifying and prioritizing various risks influencing time and cost performance of modular construction projects in Canada using Analytic Hierarchy Process (AHP) and simulation [69]. The limitations of the study are its specificity to Canadian construction industry as well as narrow focus on project objectives (time and cost). Another study identified and prioritized risks in IBS implementation in China [65]. A similar study was carried out in Malaysian construction industry context for identification and prioritization of risk factors [74, 75]. Both studies being region specific and focusing more on barriers than risks depict a limitation in studies. A study focused on cost elevation risk factors in korean modular construction industry [64]. However, the scope of this study was also limited to Korea, hence not applicable in other countries. In the quest of a decision support system for identifying, prioritizing and mitigating risk factors prevalent in modular construction, a research identified and prioritized 29 risk factors using fuzzy synthetic modelling [76]. However, the research generalized the risks and it is imperative to understand the sensitivity of risk factors based on objectives, locations, project types and countries. Hence, bespoke research studies are essential in determining the critical nature of risk factors specific to a project or location. A study modelled the risk factors in the design phase of modular construction using experts reviews from international MIC experts and evaluated the data using statistical analysis [77]. However, the study was limited to the design phase and did not take into account the construction, transportation and assembly related risk factors. Similarly, another research by Wuni et al prioritized critical risks in the assembly phase of modular construction projects by introducing an index for computing risk severity [78]. The research gave important insights into the onsite assembly related risk factors; however, the research could not take into account the other phases of modular construction project. Moreover, risk factors prevalent during the production phase of modular construction were also identified in a research study [79]. Though the study signified risks in an important supply chain function, it fails to take into account the risk factors in other segments of the modular construction supply chain. A comprehensive and systematic literature review on modular constructions risks was carried out which identified and prioritized critical risks [60]. These risks were generic meaning that they are applicable in various geographic context but the prioritization was performed using frequency of occurance. The study lacks a detailed evaluation and ranking of risks based on expertise and knowledge of industry practitioners.

After a detailed evaluation of previous literature in modular construction projects, it can be seen that the research studies are either focused on a specific phase of modular construction or the identified risk factors are generic and require bespoke studies considering region, location, project objectives. There is also a need of a comprehensive risk assessment framework that is applicable across multiple offsite construction projects. Moreover, no existing study up to date has evaluated modular construction related risks in the Pakistani construction industry. Hence, this study will provide a comprehensive framework for identification, evaluation and prioritization of critical risks in modular construction implementation in Pakistan.

Table 1 below enlists the critical risk factors shortlisted after a rigorous literature review and experts’ analysis. The entire process of shortlisting and selection of risk factors has been explained in the subsequent section.

**Table 1:** Critical risks associated with the implementation of modular construction.

S. No	Risk Factor	Reference
1.	High initial investment	[6, 59, 62, 66]
2.	Difficulty in attaining return on initial investment and longer break-even period	[49, 65, 71]
3.	Poor coordination among multi-interface	[6, 57, 80]
4.	Complex Supply chain and Stakeholder composition	[9, 26, 68, 81]
5.	Inability to make changes in design during the construction stage	[8, 45, 55, 65, 82]
6.	Change orders due to defective design	[60, 64, 69, 70]
7.	Lack of appropriate design codes and standards	[65, 71, 83]
8.	Poor government support and restrictive regulations	[35, 60, 67]
9.	Poor supply chain integration	[45, 60, 84, 85]
10.	Inadequate skills and experience in modular construction	[24, 61, 66]
11.	Skepticism and conservative attitude of terminal user	[55, 58, 65]
12.	Transportation restraints	[39, 59, 68, 86]
13.	Inept Scheduling	[26, 27, 57, 60]
14.	Lack of quality monitoring systems	[63, 65]
15.	Requirement of skilled labour	[68, 71, 81]
16.	Damage of modular components during transportation to building site and installation	[18, 46, 65]
17.	Complexity of modular building design	[34, 59, 60, 70]
18.	Technology incompetence	[46, 65]
19.	Delay in modules delivery to building site	[27, 83, 85]
20.	Inadequate capacity of modular manufacturers	[35, 45, 60, 66]

## Research methodology

This study uses a combination of qualitative and quantitative methods to identify and prioritize the critical risks arising in the implementation of offsite modular construction. A combination of both qualitative and quantitative research methodologies can prove vital in gaining insights and generating reliable and valuable results as suggested by [87]. The first step involves a detailed review of peer-reviewed articles in international journals for identifying the risks associated with the implementation of modular construction. The identified risks from literature are then submitted to a panel of experts for validation of risks in Pakistani context. Since there are various risks involved in construction projects and not all of them are critical; it requires a shortlisting of the most significant risks for further evaluation. For this purpose, group consensus can be gained from experts regarding the most critical risk factors. Fuzzy Delphi method is used in this study for shortlisting the most significant risk factors through group decision. Finally, the most critical risks are then prioritized using a novel Full Consistency Method (FUCOM). A criticality index for each risk is calculated which is equal to the priority weight obtained from FUCOM analysis and the risks are then prioritized on the basis of highest to lowest criticality index. The description of each methodological step is further illustrated in the following sections below.

### Step 1: Identification of modular construction risks from literature review

The first step involved a systematic literature search for identifying critical risks in modular construction targeting only English language peer-reviewed articles in international journals. Construction Engineering and Management (CEM) databases included in this search are Scopus, Web of Science, Elsevier, ASCE library, Emerald insight, and Taylor & Francis. The keywords used for the retrieval of relevant articles are: “risks”, “barriers”, “offsite construction” and “modular construction”. The search results generated a list of more than 120 articles in more than 30 journals. The articles were then checked for duplication and repetition of papers, after which the abstracts and titles of resultant papers were examined to ensure that the content of the articles was relevant to the objectives and scope of our research. The final list of papers selected for this study are 35 articles in 22 journals. The top three journals with most research articles are: Journal of Cleaner Production (8), Construction Management and Economics (5) and Automation in construction (3). After a detailed evaluation of previous literature, no relevant study was found in the context of Pakistan. Therefore, this study relies on generic risk factors that are specific to modular construction highlighted in previous studies. These risk factors are then subjected to a validation process to confirm the applicability of identified risks in a particular geographic context.

In the preliminary stage, a total of 24 risks were identified and a questionnaire was developed which was then scrutinized through discussions with a panel of experts for further improvements in the list of identified risks considering the background of Pakistani construction industry. Expert sampling, a subset of purposive sampling was used for shortlisting the audience for research. Relevant work experience of greater than or equal to five years was considered as the inclusion criteria for shortlisting of the experts for study. Large-scale implementation of modular offsite construction is not prevalent in Pakistan; therefore, the experts were relatively limited. The initial panel comprised of 3 experts from industry, all working in various offsite prefab and modular construction companies and 2 experts from academia having research on offsite construction. Moreover, all experts have more than 5 years of experience which is an important criterion for the selection of experts. The experts provided positive and valuable feedback regarding the questionnaire design and suggested minor improvements in the questionnaire. After the validation process, a total of 20 risk factors were finalized for further analysis. The list of finalized risk factors is shown in Table 1.

### Step 2: Fuzzy Delphi method

The classical Delphi method was introduced by Dalkey and Helmer [88] which is a survey method based on opinions from a group of experts through an iterative and controlled feedback process until all experts agree on a single point or decision under consideration [89]. However, in real life scenario, the expert opinions in Delphi method cannot be precisely converted and interpreted in numerical values [89]. Moreover, the modelling of real-life situations using crisp numerical values is insufficient due to the uncertainty, imprecision, vagueness and subjectivity involved in human judgements [90]. To encounter this issue, Fuzzy set theory proposed by Zadeh [91] was integrated with Delphi method to develop a new Fuzzy Delphi method (FDM) to improve the outcome in terms of better precision and reduced uncertainty in the decision making [92].

Previous studies in risk assessment have utilized FDM in the identification of critical risks in different contexts [93–96]. A research study made use of the FDM in barriers to sustainable practices in solid waste management [97]. The methodology utilized qualitative information obtained from waste management experts and shortlisted 44 critical barriers out of 146 barriers to sustainable practices. Another study identified critical risks in worn-out building structures during an earthquake and ranked the risks according to their probability of occurrence utilizing FDM [98]. During the outbreak of COVID-19, a research in India identified critical safety risk factors arising in hospitals among health workers [99]. The research used FDM to identify 15 safety risks whereas the hospitals were ranked based on those risk factors using a combination of Fuzzy AHP and Fuzzy TOPSIS. Researchers have also used FDM in risk management of supply chains. One such study modelled risks arising in different segments of Halal supply chain using a combination of FDM and DEMATEL approach [100]. The risks were identified via FDM whereas the prioritization as well as cause-effect study was carried out via DEMATEL to study interrelationship among risks. A similar study used FDM for the purpose of identifying factors that are critical in supplier selection considering a green sustainable supply chain [101]. The research used qualitative information in the form of expert opinion and converting it into numerical form, shortlisting 25 essential factors out of 58. Another research evaluated risks in telecommunication industry of India, identifying most important risks via literature review and FDM, whereas Interpretive Structural Modelling (ISM) was used in combination with FDM to analysis the interrelationship among critical risks [102]. FDM has also been used in the construction industry perspective, a study highlighted major risks arising on construction sites using FDM in combination with DEMATEL to examine cause and effect of risk factors [103]. A similar research examined the occupational health and safety (OHS) risks associated with green building construction projects [104]. The study employed FDM in combination with Best-Worst method (BWM) in order to enlist and rank safety risk factors. Also, a research identified 32 risks from Project Management Body of Knowledge (PMBOK) and finalized 17 critical risk factors via Fuzzy Delphi method to develop a hybrid qualitative and quantitative risk evaluation framework under uncertainty [105]. A study in offsite construction perspective developed a risk assessment model for identifying and ranking risks arising in the implementation of Building Information Modelling (BIM) in modular construction projects [106]. The research combined FDM with DEMATEL approach to classify and rank risk factors in BIM implementation in offsite modular construction projects.

Considering the applications of FDM in risk assessment as well as construction industry, this research recommends the selection of FDM as a suitable method for risk assessment of modular construction projects. The steps involved in the FDM are given below:

**1**. **Identification of the possible risks relevant to this study**
  In this study, the possible risks pertaining to the implementation of modular construction were identified through literature review and validation of experts as shown in Table 1.
**2**. **Collection of opinions from experts**
  After the identification of risks, a suitable number of experts are invited and asked to determine the importance of a particular risk factor based on its impact on the implementation of modular construction. A questionnaire is used to obtain the responses of experts in the form of linguistic variables such as ‘Very High’ ‘Very Low’ etc. These linguistic variables are then converted into corresponding fuzzy numbers using a scale shown in Table 2.

**Table 2:** Linguistic scale.

Linguistic variable	Fuzzy number
Very Low	(0, 0, 0.1)
Low	(0, 0.1, 0.3)
Medium Low	(0.1, 0.3, 0.5)
Medium	(0.3, 0.5, 0.7)
Medium High	(0.5, 0.7, 0.9)
High	(0.7, 0.9, 1.0)
Very High	(0.9, 1.0, 1.0)

Source: [107]
  Moreover, triangular fuzzy sets have been used to covert linguistic variables in numeric form.
**3**. **Determination of experts’ group decision weights**
  To determine the experts’ group decision, the model developed by Hsu et al [89] has been used in this study.
  The formula for converting individual responses into a group decision is described below:
  If the significance of a risk factor ‘m’ assigned by expert ‘l’ of ‘n’ experts is D_lm_ = (a_lm_, b_lm_, c_lm_) where (l = 1,2,3,…,n) and (m = 1,2,3…,k), The fuzzy number corresponding to group decision of risk factor ‘m’ is D_m_ = (a_m_, b_m_, c_m_) where (m = 1,2,3…,k) among which,
$${\text{a}}_{\text{m}}=Min_{l}\;\left\{a_{lm}\right\}$$ (1)
$${\text{b}}_{\text{m}}=\frac{1}{n}\sum_{l=1}^{n}b_{\mathrm{lm}}$$ (2)
$${\text{c}}_{\text{m}}=Max_{\text{l}}\;\left\{c_{lm}\right\}$$ (3)
**4**. **Defuzzification**
  The weights obtained from step 3 for each risk factor are defuzzified using center of gravity method to obtain crisp values S_m_ for each risk. The formula for defuzzification is given below:
$${\text{S}}_{\text{m}}=\frac{am+bm+cm}{3},\left(\text{m}=1,2,3\ldots ,\;\text{k}\right)$$ (4)
**5**. **Identification of important risk factors**
  Finally, the most important risks are determined through a comparison of crisp values of each risk factor obtained from step 4 with a threshold value “μ”. The value of μ is calculated by taking the average of weights of all risk factors’ S_m_ weight. The following criteria is used for the selection and rejection of a risk for further analysis:
  If S_m_ ≥ μ  then the risk factor “m” is selected
  If S_m_ < μ  then the risk factor “m” is rejected

### Step 3: Full Consistency Method (FUCOM)

The FUCOM is a novel multi-criteria decision-making (MCDM) method developed by Pamučar, Stević, and Sremac [108] for determination of criteria weights through pairwise comparison of criteria. FUCOM has many applications and has been applied by researchers in recent research studies. A research determined and prioritized the driving factors associated with cryptocurrency investments using a FUCOM-FB model [109]. The methodology developed a comprehensive decision support framework that can be utilized by investors, policy makers and regulators in block chain technology implementation. A research used Fuzzy FUCOM model in the ranking of demand management measures in transportation system, considering Istanbul urban mobility system as a case in point [110]. Under uncertain conditions, the model helped managers in assessing the demand management measures based on pairwise comparisons generating accurate and credible results. Fuzzy FUCOM in combination with Fuzzy weighted sum method (WSM) was used for developing a sustainability framework in farm tourism, whereby Fuzzy FUCOM allocated priority weights to important sustainability indices whereas Fuzzy WSM calculated the composite score [111]. The utilization of proposed methodology generated sustainability indices, that can be utilized for contrasting different sites’ performance, recognizing critical hotspots and benchmarking for best practices. A similar research utilized a hybrid Fuzzy MCDM framework for sustainability assessment of sewage sludge-to-energy situation [112]. Fuzzy FUCOM was used to perform pairwise comparison of important criteria whereas GRA, PRSRV, TOPSIS techniques ranked best alternatives under the scenario. Another study identified cold supply chain resilience strategies to combat COVID-19 via literature review and ranked the strategies used a fuzzy FUCOM model [113]. The results of study gave important insights to top most effective strategies for tackling risks associated with COVID-19 in a cold supply chain. In a recent study, a hybrid Fuzzy FUCOM-QFD model was implemented in the prioritization of resilient strategies to combat the spread of COVID-19 in Pakistan’s healthcare sector [114]. The study developed a comprehensive framework that can be used by policy makers and health practitioners in determining the best strategies to combat pandemics in the healthcare sector. A hybrid Fuzzy FUCOM-VIKOR-QFD has also been utilized in developing a risk assessment framework for electric power sector of Pakistan [115, 116]. The criteria weight coefficients were determined by Fuzzy FUCOM whereas Fuzzy VIKOR ranked the risks according to the weight coefficients obtained from FUCOM. The mitigation strategies for risks were then prioritized using a Fuzzy-QFD model. The hybrid Fuzzy MCDM model provided accurate results under uncertain conditions.

The applications of FUCOM in various research areas make it a suitable methodology for implementation in the current research. There are other similar MCDM methods similar to FUCOM such as AHP and BWM that evaluate factors based on pairwise comparisons. According to a research study [109], Analytic Hierarchy Process (AHP) is most widely used as a risk assessment tool for prioritization of construction risks. However, this method suffers from drawbacks such as too many pairwise comparisons and redundancy in comparison of risk factors, causing errors in judgements. This problem is effectively addressed in the novel FUCOM method such that it requires less pairwise comparisons equal to n-1 where “n” is the number of risks as criteria and also sets certain constraints for the determination of optimal values of risk factors weights [108].

Also, comparing BWM with FUCOM, BWM requires a pair of comparison vectors for evaluation of criteria, whereas FUCOM only requires one vector that results on the reduction in number of comparisons. Moreover, FUCOM also calculates an error value DFC (deviation from full consistency) for the criteria weight, thereby providing a validation of the model [117]. Taking into consideration the advantages of FUCOM over other MCDM methods including AHP and BWM, this research justifies the selection of FUCOM for risk assessment of modular construction projects in Pakistan.

In this study, the critical risks in modular construction are considered as ‘criteria’ in FUCOM and the weights of criteria will denote the criticality index of risk factors. The steps involved in the methodology are described below:

**1**. **Ranking of criteria**
  The first step involves the ranking of criteria according to the level of significance of each criterion. The ranking is performed by a group of experts who possess extensive knowledge and experience in their respective field. Considering the critical risks as criteria, the set of risks *CR* = {*CR*_1_,*CR*_2_,*CR*_3_,…,*CR*_*n*_} sare then arranged in the increasing order of their rank as follows:
$$CR_{j\;\left(1\right)}>CR_{j\left(2\right)}>CR_{j\left(3\right)}\ldots >CR_{j\left(R\right)}$$ (5)
  Where R denotes the rank of the observed risk factor. If two risk factors have the same level of significance, then the “>” sign is replaced by “=“ in expression (5).
**2**. **Determination of comparative priority**
  Comparative priority can be defined as the advantage of criterion/risk CR_j(R)_ rank over criteria/risk CR_j(R+1)_ rank. There are two methods for the calculation of comparative priorities i.e. the relative advantage of risk factor CR_j(R)_ over risk factor CR_j(R+1)_.
  - The first method deals with precise values where criteria/risks have absolute values/or weights. In this case the comparative priority ${ȹ}_{\frac{R}{R+1}}$ of criteria/risk CR_j(R)_ over CR_j(R+1)_ is calculated by dividing the absolute value/weight of criteria/risk CR_j(R)_ by the absolute value of criteria/risk CR_j(R+1)_. In this method, the decision makers compare the criteria/risks on the basis of their internal knowledge, hence the comparative priorities are computed based on subjective preference.
  - The second method allows the use of a pre-defined scale when precise values for criteria cannot be determined. In this case, the decision makers first compute the significance of each criterion ω_R_ in expression (5) by comparing all the criteria, one-by-one with the most significant (top ranked) criteria. The significance of top-ranked criterion with respect to itself will be equal to 1. The significance of criteria is computed via a predefined scale i.e. ω_R_ ∈ [1,9]. On the basis of significance of criteria and equation $\frac{\omega_{R}}{\omega_{R+1}}=\;{ȹ}_{\frac{R}{R+1}}$ the comparative priorities can be calculated.
  Hence the comparative priority vectors of all criteria are obtained as shown below:
$$ȹ=\left({ȹ}_{\frac{1}{2}},\;{ȹ}_{\frac{2}{3}},\;{ȹ}_{\frac{3}{4},}\ldots ,\;{ȹ}_{\frac{R}{R+1}}\right)$$ (6)
**3**. **Calculation of weight coefficients of criteria**
  In this step, the weight coefficient values of criteria are calculated (w_1_, w_2_, w_3_, …, w_n_) ^T^. The following two conditions must be satisfied for accepting the values of weight coefficients.
  **Condition 1:** The ratio of weight coefficients must be equal to the comparative priority of the criteria under observation $\left({ȹ}_{\frac{R}{R+1}}\right)$ i.e.
$$\frac{w_{R}}{w_{R+1}}={ȹ}_{\frac{R}{R+1}}$$ (7)
  **Condition 2:** The condition of mathematical transitivity must be satisfied among the values of criteria weights i.e.
$$\frac{w_{R}}{w_{R+2}}=\;{ȹ}_{\frac{R}{R+1}}\;⊗\;{ȹ}_{\frac{R+1}{R+2}}$$ (8)
  Based on Eqs (7) and (8), the final model for determination of criteria weights can be written as follows:
minχ
  s.t.
$$\left|\begin{array}{c} \frac{w_{j\left(R\right)}}{w_{j\left(R+1\right)}}-\;{ȹ}_{\frac{R}{R+1}} \end{array}\right|\;\leq \;\chi ,\;\forall_{j}$$
$$\left|\frac{w_{R}}{w_{\left(R+2\right)}}\;-\;{ȹ}_{\left(\frac{R}{R+1}\right)}⊗\;{ȹ}_{\frac{R+1}{R+2}}\right|\leq \;\chi ,\;\forall_{j}$$ (9)
$$\sum_{j=1}^{n}w_{j}=1\;,\;\forall_{j}$$
$$w_{j}\geq 0,\;\forall_{j}$$
  Where *χ* denotes the minimum DFC value, which is satisfied only if the transitivity is fully respected.
  The solution of expression (9) in a linear programming solver results in the calculation of final values of criteria weights (w_1_, w_2_, w_3_, …, w_n_) ^T^ and the DFC (*χ*) is also generated.

### Modified FUCOM method

Conventional FUCOM method relies on the input from decision makers where in the first stage, decision makers rank the criteria followed by their relative comparison in the second stage. This method, though having less pairwise comparisons than AHP and BWM method, is still exhaustive and time consuming since each decision maker may have his own preference regarding the criteria thereby requiring separate analysis for each decision maker. Instead of performing separate questionnaire surveys from decision makers in FDM and FUCOM method, this study suggests the continuation of FUCOM taking the input from FDM for its initial step i.e. the ranking of criteria. The essential risk factors obtained from FDM are rearranged in descending order of their S_m_ value and ranked accordingly. This rank on the basis of S_m_ value which represents ‘consensus of decision makers’ in FDM is used in FUCOM instead of individual ranking by decision makers. This eliminates the need for performing separate survey for FUCOM since the essential risks in FDM are obtained from the consensus of all experts. A similar approach has been used in a study where rank from Delphi method was utilized in the FUCOM for its first step [118].

The next step involves the pairwise comparison of all risk factors to obtain the comparative priority. Here, instead of relying on experts for performing pairwise comparisons individually, the S_m_ values can be used for this purpose. Followed by ranking of risk factors on the basis of S_m_ value in Step 1, the significance of each risk factor is calculated by dividing the S_m_ value of top ranked risk with S_m_ value of the successor risk factor. Subsequently, the comparative priority of each risk factor is calculated as described in Step 2b of FUCOM method. The remaining steps are similar to the conventional FUCOM method.

## Analysis and results

The risk factors identified from literature review (shown in Table 1) are evaluated for highlighting the most essential risk factors for prioritization. A questionnaire survey included all these risks and a seven-point Likert scale was used to ascertain the significance of each risk according to decision makers. The survey questionnaire was sent to experts having knowledge and experience in offsite prefab and modular construction through various platforms including ResearchGate, LinkedIn, companies and academic institution websites. The questionnaire included basic demographic information such as qualification, name of the company/institution, designation, years of experience in offsite construction etc. The sampling method, inclusion and exclusion criteria for selecting survey respondents were the same as implemented in the literature review stage for shortlisting risk factors. A total of 45 questionnaires were sent out of which, only 15 valid responses were obtained. The suitable number of responses for FDM lie in the range of 10–30 as depicted by previous studies [89, 107, 119]. Hence, a sample of 15 respondents can be considered appropriate for the analysis. The profile of experts is shown in Table 3.

**Table 3:** Profile of experts.

Group	Qualification	Position/Designation	Years of Offsite construction experience
Contractor	Bachelors	Construction Manager	5
	Masters	Project Manager	10
	Bachelors	Resident Engineer	7
	Bachelors	Project Manager	15
	Masters	Department Manager	6
	Masters	Engineer	5
	Masters	Project Manager	8
	Bachelors	Engineer	5
	Bachelors	Construction Manager	10
	Bachelors	Project Manager	8
Client	Masters	Manager	10
	Masters	Manager	8
Academic	PhD	Professor	6
	PhD	Assistant Professor	5
	PhD	Assistant Professor	5

The experts were asked to highlight the significance of a risk based on its effect on the implementation of modular construction. The responses from experts are then used in Step 2 i.e. Fuzzy Delphi analysis. The results of FDM are described in Table 4:

**Table 4:** Results of Fuzzy Delphi method.

Critical Risk number CR	Risk Factor	S_m_ value	Selected/Rejected
1.	High initial investment	0.582	Accepted
2.	Difficulty in attaining return on initial investment and longer break-even period	0.54	Accepted
3.	Poor coordination among multi-interface	0.531	Rejected
4.	Complex Supply chain and Stakeholder composition	0.558	Accepted
5.	Inability to make changes in design during the construction stage	0.609	Accepted
6.	Change orders due to defective design	0.511	Rejected
7.	Lack of appropriate design codes and standards	0.531	Rejected
8.	Poor government support and restrictive regulations	0.529	Rejected
9.	Poor supply chain integration	0.529	Rejected
10.	Inadequate skills and experience in modular construction	0.622	Accepted
11.	Skepticism and conservative attitude of terminal user	0.544	Accepted
12.	Transportation restraints	0.604	Accepted
13.	Inept Scheduling	0.471	Rejected
14.	Lack of quality monitoring systems	0.487	Rejected
15.	Requirement of skilled labour	0.562	Accepted
16.	Damage of modular components during transportation to building site and installation	0.556	Accepted
17.	Complexity of modular building design	0.509	Rejected
18.	Technology incompetence	0.556	Accepted
19.	Delay in modules delivery to building site	0.549	Accepted
20.	Inadequate capacity of modular manufacturers	0.611	Accepted
	**Threshold Value**	**0.54**	

The risk factors excluded from the analysis include ‘Poor coordination among multi-interface’, ‘Change orders due to defective design’, ‘Lack of appropriate design codes and standards’, ‘Poor government support and restrictive regulations’, ‘Poor supply chain integration, ‘Inept scheduling’, ‘Lack of quality monitoring systems’ and ‘Complexity of modular building design’. These risk factors have a group decision weight S_m_ less than the threshold value, which means that the experts did not consider these factors significant. Hence, the most critical risk factors have been highlighted by FDM which will be used for prioritization and calculation of the criticality index. After removal of non-significant risk factors, the ranking based on FDM in the decreasing order of S_m_ value is shown below in Table 5.

**Table 5:** Rank of critical risks based on group decision weight.

Critical Risk number CR	Risk Factor	S_m_ value	Rank
CR_1_	Inadequate skills and experience in modular construction	0.622	1^st^
CR_2_	Inadequate capacity of modular manufacturers	0.611	2^nd^
CR3	Inability to make changes in design during the construction stage	0.609	3^rd^
CR4	Transportation restraints	0.604	4^th^
CR5	High initial investment	0.582	5^th^
CR6	Requirement of skilled labour	0.562	6^th^
CR7	Complex Supply chain and Stakeholder composition	0.558	7^th^
CR8	Damage of modular components during transportation to building site and installation	0.556	8^th^
CR9	Technology incompetence	0.556	9^th^
CR_10_	Delay in modules delivery to building site	0.549	10^th^
CR_11_	Skepticism and conservative attitude of terminal user	0.544	11^th^
CR_12_	Difficulty in attaining return on initial investment and longer break-even period	0.54	12^th^

The significance of using the FDM is that it shortlists the most important risk factors that have a relatively high impact on the implementation of modular construction and rejects those risks which do not have significant impact on the implementation of modular construction. Moreover, in this study, FDM provides the group decision weights and rank which is further used in FUCOM for determination of criticality index.

Modified FUCOM was applied in the next step to prioritize the risk factors and to calculate the criticality index. Firstly, the 12 critical risks shortlisted from FDM analysis are ranked on the basis of their group decision weight i.e. S_m_ as shown in Table 5. in which CR_1_, CR_2_, CR_3_, …, CR_12_ represents the rank of 12 risk factors on the basis of S_m_ value. Then the significance of each risk factor is calculated by comparing each risk with the top-rank risk i.e. CR_1_. For example, the significance of the top ranked risk i.e. CR_1_ with respect to itself is ω_1_ = 1. Similarly, the significance of CR_2_ will be ω_2_ = S_1_/ S_2_ = 1.018. Similarly, the significance of all other risk factors is calculated. The next step calculates the comparative priorities of the ranked risk factors. For example, the significance ω_1_ and ω_2_ of the risk factor CR_1_ and CR_2_ is 1 and 1.018 respectively, Using the equation $\frac{\omega_{R}}{\omega_{R+1}}=\;{ȹ}_{\frac{R}{R+1}}$ the comparative priority for CR_1_ can be calculated as $\frac{\omega_{2}}{\omega_{1}}=\frac{1.018}{1}$ which implies that ${ȹ}_{\frac{1}{2}}=\frac{1.018}{1}=1.018$. Similarly, the comparative priorities for all other risk factors are calculated as shown in Table 6 below.

**Table 6:** Comparative priorities of critical risk factors.

Comparative Priorities	
ȹ_1/2_	1.018
ȹ_2/3_	1.003
ȹ_3/4_	1.008
ȹ_4/5_	1.038
ȹ_5/6_	1.036
ȹ_6/7_	1.007
ȹ_7/8_	1.004
ȹ_8/9_	1.000
ȹ_9/10_	1.013
ȹ_10/11_	1.009
ȹ_11/12_	1.007

Finally, the criticality index i.e. weight coefficients of all risk factors (w_1_, w_2_, w_3_, …, w_n_) ^T^ are calculated which must satisfy the conditions in Eqs (7) and (8). Applying the first condition i.e. Eq (7) yields the following expression:

$$w_{1}/w_{2}=1.018,\;w_{2}/w_{3}=1.003,\;w_{3}/w_{4}=1.008,\;w_{4}/w_{5}=1.038,\;w_{5}/w_{6}=1.036,\;w_{6/}w_{7}=1.007,\;w_{7}/w_{8}=1.004,\;w_{8}/w_{9}=1.000,\;w_{9}/w_{10}=1.013,\;w_{10}/w_{11}=1.009,\;w_{11}/w_{12}=1.007$$ (10)

Similarly, after applying section condition i.e. Eq (8) yields the following expression:

$$w_{1}/w_{3}={ȹ}_{1/2}\times {ȹ}_{2/3}=1.021,\;w_{2}/w_{4}={ȹ}_{2/3}\times {ȹ}_{3/4}=1.012,\;w_{3}/w_{5}={ȹ}_{3/4}\times {ȹ}_{4/5}=1.046,\;w_{4}/w_{6}={ȹ}_{4/5}\times {ȹ}_{5/6}=1.075,\;w_{5}/w_{7}={ȹ}_{5/6}\times {ȹ}_{6/7}=1.043,\;w_{6/}w_{8}={ȹ}_{6/7}\times {ȹ}_{7/8}=1.011,\;w_{7}/w_{9}={ȹ}_{7/8}\times {ȹ}_{8/9}=1.004,\;w_{8}/w_{10}={ȹ}_{8/9}\times {ȹ}_{9/10}=1.013,\;w_{9}/w_{11}={ȹ}_{9/10}\times {ȹ}_{10/11}=1.022,\;w_{10}/w_{12}={ȹ}_{10/11}\times {ȹ}_{11/12}=1.017$$ (11)

The results from expression (10) and (11) can be used to generate a non-linear optimization model for determining deviation from full consistency i.e. DFC (*χ*) to represent the error in weights obtained. The final equation for calculating the weights of risk factors is given below:

Min=χ,

Subjected to

$$\begin{array}{l} |\frac{w_{1}}{w_{2}}-1.018|\leq \;\chi \;,|\frac{w_{2}}{w_{3}}-1.003|\leq \;\chi ,|\frac{w_{3}}{w_{4}}-1.008|\leq \;\chi ,|\frac{w_{4}}{w_{5}}-1.038|\leq \;\chi ,|\frac{w_{5}}{w_{6}}-1.036|\leq \\ \chi ,|\frac{w_{6}}{w_{7}}-1.007|\leq \;\chi ,|\frac{w_{7}}{w_{8}}-1.004|\leq \;\chi ,|\frac{w_{8}}{w_{9}}-1.000|\leq \;\chi ,|\frac{w_{9}}{w_{10}}-1.013|\leq \;\chi ,|\frac{w_{10}}{w_{11}}-1.009|\\ \leq \;\chi ,|\frac{w_{11}}{w_{12}}-1.007|\;\leq \;\chi ,\;|\frac{w_{1}}{w_{3}}-1.021|\leq \;\chi ,|\frac{w_{2}}{w_{4}}-1.012|\leq \;\chi ,|\frac{w_{3}}{w_{5}}-1.046|\leq \chi ,|\frac{w_{4}}{w_{6}}-1.075|\leq \;\chi ,|\frac{w_{5}}{w_{7}}-1.043|\leq \;\chi ,|\frac{w_{6}}{w_{8}}-1.011|\leq \;\chi ,|\frac{w_{7}}{w_{9}}-1.004|\leq \;\chi ,|\frac{w_{8}}{w_{10}}-1.013|\\ \leq \;\chi ,|\frac{w_{9}}{w_{11}}-1.022|\leq \;\chi ,|\frac{w_{10}}{w_{12}}-1.017|\leq \;\chi ,\;w_{1}+w_{2}+w_{3}+w_{4}+w_{5}+w_{6}+w_{7}+w_{8}+w_{9}+w_{10}+w_{11}+w_{12}=1 \end{array}$$ (12)

**Fig 1:**
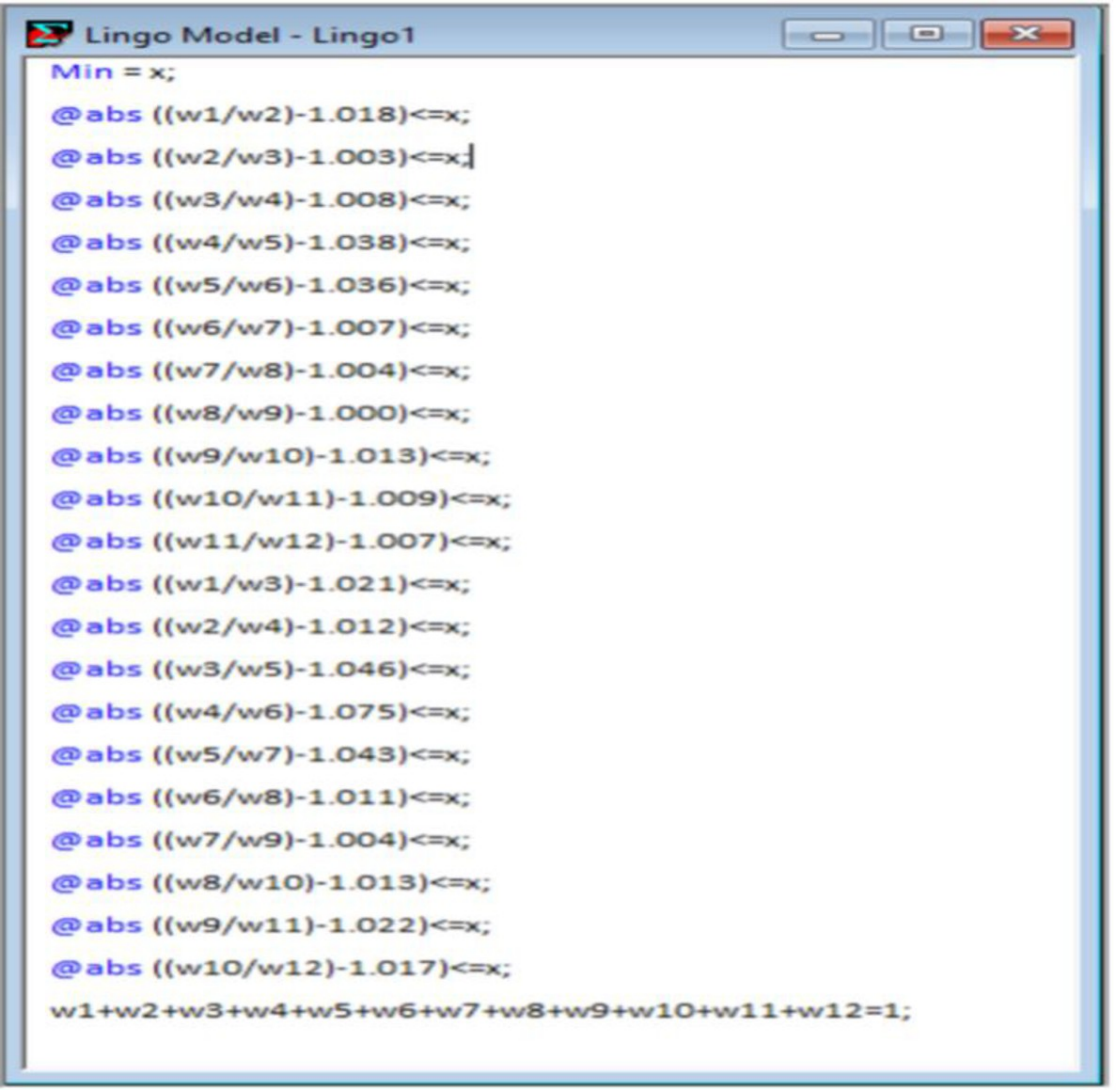
Code description on LINGO software.

**Fig 2:**
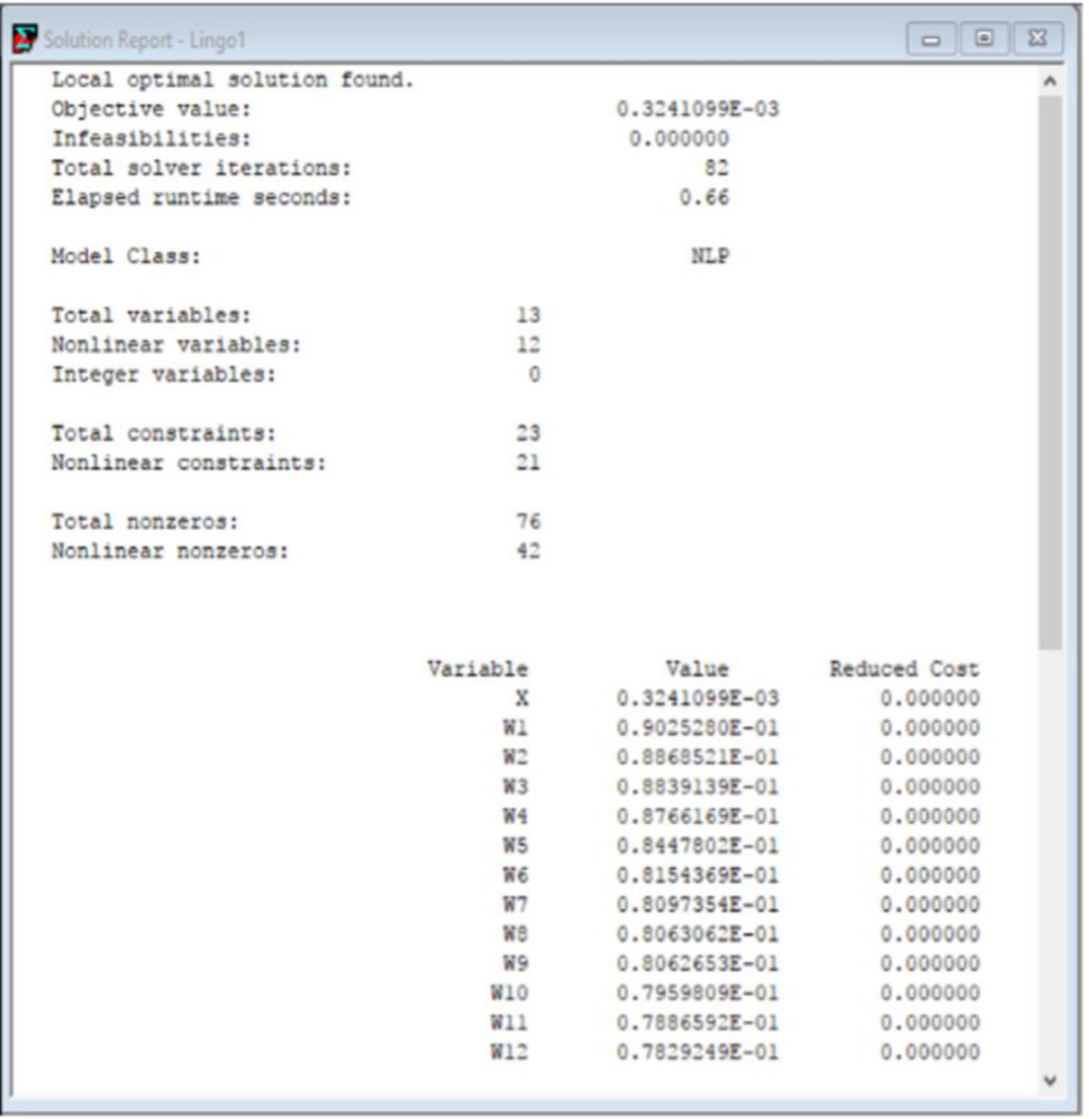
Optimal solution obtained from LINGO software.

After solving the above expression in LINGO software version 18.0, the final weight coefficients i.e. the criticality index of each risk factor is obtained. The program code as well as the optimal solution resulting from the software are given in Figs 1 and 2 respectively.

The criticality index of each risk factor is described in Table 7.

**Table 7:** Final ranking of critical risks based on criticality index.

Critical Risk number CR	Risk Factor	Criticality index	Rank
CR_1_	Inadequate skills and experience in modular construction	0.0902	1^st^
CR_2_	Inadequate capacity of modular manufacturers	0.0886	2^nd^
CR3	Inability to make changes in design during the construction stage	0.0884	3^rd^
CR4	Transportation restraints	0.0877	4^th^
CR5	High initial investment	0.0845	5^th^
CR6	Requirement of skilled labour	0.0815	6^th^
CR7	Complex Supply chain and Stakeholder composition	0.0809	7^th^
CR8	Damage of modular components during transportation to building site and installation	0.0806	8^th^
CR9	Technology incompetence	0.0806	9^th^
CR_10_	Delay in modules delivery to building site	0.0796	10^th^
CR_11_	Skepticism and conservative attitude of terminal user	0.0789	11^th^
CR_12_	Difficulty in attaining return on initial investment and longer break-even period	0.0782	12^th^

It is important to mention that the rank obtained from FUCOM in Table 7 is similar to FDM since the ranking of risk factors in the first step of FUCOM is obtained from FDM which is subsequently used in the later steps. However, the rationale of using FUCOM is as follows:

- FDM results in the ranking of factors based on absolute weights whereas FUCOM computes the weight coefficients and ranking of risk factors based on pairwise comparison.
- FUCOM provides full consistency in a sense that it uses the consensus of decision makers from FDM and consequently performs pairwise comparisons of most essential risk factors based on the group decision weight i.e. S_m_ value, thereby eliminating the need for additional survey from experts for pairwise comparison.
- The weight coefficients i.e. criticality index obtained from FUCOM is free from any inconsistency since the method validates the computed weights as well as ranking based on two conditions as mentioned in Eqs (7) and (8). Moreover, the sum of weights obtained from FUCOM analysis are equal to 1 in this study which also validates that the computed weight coefficients are consistent and obeying the steps of methodology.
- An error term *χ* is also calculated which determines the error in weight coefficients and it must be closer to zero. This value equals 0.000324 in this study, suggesting that the analysis and obtained weight coefficients are correct.

## Discussion

Table 7 shows the final ranking and criticality index of risk factors. It can be seen that the most significant risk in the implementation of modular construction in Pakistan is ‘Inadequate skills and experience in modular construction’ having the highest criticality index i.e. 0.0902. This risk has also been highlighted in a study by [66] who specifically mentioned that inadequate skills and experience in modular construction is more prevalent in developing countries. Modular construction involves many stakeholders and consists of multiple segments which requires efficient and effective coordination among the involved parties. Inexperience in modular construction projects can ultimately lead to disruption in schedule performance resulting in delayed deliveries and reduced quality. The prominent reason for the lack of skills and experience is the innovative nature of the offsite modular construction in Pakistani construction industry. Even through offsite modular construction is currently being used for meeting the housing needs of society, the rate of adoption is relatively lower as compared to other countries such as China and Malaysia. Some of the noteworthy construction firms using prefabricated and modular construction techniques in Pakistan are Frontier Works Organization (FWO), ModulusTech, Izhar Foster, Paragon Constructors and Majestic builders. Since the relative adoption of modular construction is slower than other countries, Pakistani contractors are less experienced in managing modular construction projects. This can also be supported from the fact that no mega-construction project has yet been carried out utilizing modular construction. Moreover, there is a lack of continuous improvement and innovation culture in Pakistan, due to which contractors mostly rely on conventional skills and methods and show reluctance towards innovation and learning new skills. The consequences of having less skills and experience are the highest since it can lead to defective design, poor facility management and production, poor coordination, poor quality and substandard erection practices. Since modular construction supply chain is highly fragmented and interdependent, lack of expertise can result in dissention between manufacturers and designers in the initial stages of construction, problems during the production phase and delay in delivery of modular components to site. The way forward to address this risk is to adopt the construction practices from other developed countries in handling mega modular construction projects. Training and skill development programs in cost-benefit analysis, design characteristics and methods can also reduce the skill gap of Pakistani contractors. In this regard, it is important to mention that the government of Pakistan is in negotiation with Chinese construction company to execute mega housing project in Pakistan utilizing modular construction [120]. This can be a significant step since local contractors can gain insights to the techniques and methods to gain skills while also gaining experience in managing mega projects.

While inadequate skills and experience in modular construction is considered critical, another important risk affecting modular construction implementation is the inadequate capacity of modular manufacturers, which is the second most critical risk with a criticality index 0.0886. Generally, the production line for manufacturing of a typical modular house consists of 20–24 manufacturing stations. The decision regarding the fabrication of a single or double-unit module depends on the size of manufacturing facility. Along with the size requirement for manufacturing stations, space for quality control checks is also required separately. Moreover, the factory requires separate storage space for finished modules before shipping these modules to the project site. Majority of offsite construction firms in Pakistan are small or medium sized where limited financial resources hinder modular manufacturers to upgrade their capacity according to the space requirements. This subsequently restricts the number as well as size of modular components to be manufactured. Due to the novelty of offsite construction in Pakistan, high rates of prefabrication and modularization are not feasible at this stage since it may lead to quality related issues due to inexperience such as defective designs, leakages and cracks propagations, geometric intolerances and joint failures etc. The risk of low production capacity can make it difficult for modular construction firms to gain market growth when demand is increased. Hence, to mitigate this risk, the prime focus should be improvement in the quality of manufacturing. Once a desired level of quality is attained, the next focus should be financing these small and medium sized firms to increase their production capacity. Modular construction firms can also collaborate with external partners that provide warehousing and storage services in case where internal expansion is relatively less feasible from an economic point of view.

Another critical risk highlighted by experts is the inability to make changes in the design during the construction stage if needed, which has been ranked third with a criticality index of 0.0884. The design process is the preliminary step in modular construction supply chain in which buildings are designed based on local building codes and standards, which requires early involvement of multiple stakeholders to give valuable input regarding the design to finalize and subsequently freeze design. This aids the design of building to freeze early, to enable the subsequent phases such as permits and approvals, material procurement, and manufacturing to commence. However, this limits the design to be modified in the later stages if any error or defect arises during the on-site assembly stage, resulting in change orders by customers. The entire process of redesign, remanufacture and reassemble then becomes time consuming, also leading to loss in efficiency. It might also require additional cost if the modifications require reconfiguration of material procurement or assembly line. The rigid nature of design process prevents the adoption of modular construction by construction firms in Pakistan since traditional construction method allows changes in the design till the last possible moment which makes it easy to readjust budget and cut costs. Existing modular construction firms in Pakistan should focus on early and effective collaboration among contractor, material supplier, manufacturer, distributor and other involved project participants to gain a consensus on the final design in order to avoid future disagreements. Furthermore, pilot projects involving fabrication of a prototype similar to the desired module and trail assembly runs must be carried out to reduce further chances of geometric variabilities and defects in design.

Another important risk factor impeding the adoption of modular construction is the transportation constraints, being ranked fourth with a criticality index of 0.0877. Since modular components have larger dimensions and are quite heavy, they require special transportation vehicles such as Self-Propelled Modular Trailer or shipping containers, for transportation to project site for final assembly. Since road transport is the most viable option for modular components transportation to site, there is a definite requirement of separate, well-constructed highways having less surface irregularity, since the components are prone to crack initiation and surface deterioration during transportation. The current highway infrastructure of Pakistan faces issues such as increased traffic congestion, deteriorated road surfaces, lack of maintenance, and negligence of government. These conditions are risky for the transportation of modular components as it can damage the components during transportation to construction site. Additionally, the highways are subjected to strict regulations regarding the size and weight of transportation vehicles, thus limiting the transportation capacity of modules. This is a significant issue as it can result in schedule delays as a bottleneck in the supply chain. Transportation related risks can be mitigated by devising lenient policies and regulations on size and weight of modules to provide assistance to modular manufactures. In addition, road infrastructure should be improved through regular maintenance, periodic check and balance, and expansion of roads and highways for accommodation of large special-purpose vehicles.

There is a significant amount of initial investment required for modular construction which includes land acquisition cost, cost of building manufacturing facilities, purchasing equipment, molds, hiring skilled labour and cost associated with the warehouses for temporary storage of modules. This risk factor is ranked fifth with a criticality index 0.0845. Despite being cost effective in the long run, the initial capital required for setting up the manufacturing of modules creates uncertainty in the mind of investors. Moreover, due to uncertain demand and monopoly of conventional construction firms in Pakistan, investors find it difficult to comprehend the potential future benefits of modular construction. Uncertainty in demand, made-to-order nature of modular components, higher prices of construction materials, and highly customized nature of the modular components also make it difficult to achieve benefits from economies of scale, consequently making it tough to attain return on initial investment. The investment related risk can effectively be mitigated through time savings, resource reduction and labour savings. It is important to mention that financial assistance is offered by the government to modular manufacturers in developed countries such as Singapore for the promotion of modular manufacturing SMEs. Considering the growing need of houses in Pakistan, similar approach can be adopted by Pakistan government to assist modular manufacturers financially to carry out large scale production of houses. In this way, housing demand can be met timely while also availing the benefits of economies of scale and reduction in per unit cost.

Other critical risks in the implementation of modular construction include requirement of skilled labour, complex stakeholder and supply chain composition, damage of modular components during transportation to building site and installation, technology incompetence, delay in modules delivery to building site, skepticism and conservative attitude of terminal user and difficulty in attaining return on initial investment and longer break-even period. The prioritization of risks provided in this study through a decision support system can help industry practitioners in identifying the most critical risks to address first along with devising appropriate strategies for successful mitigation of identified risks.

## Limitations and future recommendations

The study provides significant contribution to the theoretical body of knowledge; however, there are some limitations in the current study. First of all, the results of this study are specific to Pakistani construction industry context. Each country’s construction industry is characterized by its own set of risks and uncertainties due to different level of development. Secondly, even though the sample size of experts lies within the acceptable range, it is better to obtain more responses to better highlight the current status of modular construction implementation. Moreover, the risk factors are not categorized or grouped into well-defined dimensions in this study. Future studies can rectify the limitations of this study by categorizing risk factors based on the nature of risks in well-defined dimensions. Future studies can also try to carry out a comparative analysis of risks between developing and developed countries to highlight the differences. Since risk factors are interrelated, future studies can also model the mutual interaction among these risks through various quantitative and qualitative methodologies. Finally, the hybrid methodology used in this study relies on experts’ judgement, hence prone to subjectivity and bias. Future studies should use a case study-based approach to validate the identified risks or to generate new set of risks.

## Conclusion

The adoption of modular construction is increasing in many countries due to its well documented benefits; however, for developing countries, this method is novel and the current rate of adoption is low due to various risk events and uncertainties associated with it. Since risks are inevitable, it is necessary for practitioners to identify, evaluate, prioritize, control and monitor these risks.

- This study focused on the development of a comprehensive framework for identification and prioritization of critical risks in the implementation of modular construction in Pakistan.
- For risk prioritization, a hybrid Fuzzy Delphi and MCDM based Full Consistency Method (FUCOM) was used.
- The decision support system in this study modelled risks based on their relative importance while also resolving the issues of imprecision, uncertainty, vagueness and inconsistency through the hybrid methodology.
- The result of this study determined 12 critical risks that have a detrimental impact on the implementation of modular construction in Pakistan. The top five risks were ‘Limited skills and experience of contractors in modular construction’, ‘Inadequate capacity of modular manufacturers’, ‘Inability to make changes in design during the construction stage’, ‘Transportation restraints’ and ‘High initial investment’ respectively.
- The results of this study can be used by potential modular manufacturers while implementing an offsite construction project to look for most prominent risks and devise corresponding risk mitigation strategies.
